# Optimization for the Human Resources Management Strategy of the IoT Industry Based on AHP

**DOI:** 10.1155/2022/3514285

**Published:** 2022-05-11

**Authors:** Jingjie Wang, Wei Bai, Yongbin Liu

**Affiliations:** Hui Hua College of Hebei Normal University, Shijiazhuang, Hebei 050091, China

## Abstract

Since the twenty-first century, new things are emerging in the market economy; the Internet is one of them. Internet of things (IoT) is a new thing, which combines computer, Internet, and mobile communication network after the third wave of information industry, the development of huge application prospect, has been listed as one of the five emerging strategic industries. With the rapid development of digital technology and market economy, to accelerate the competition in the market economy, intense competition in IoT industry also exists. Effect of human resource management to a certain extent determines the strength of the market competitiveness of an enterprise; the research about human resource management of the IoT industry is carried out in this paper, by analysis of the basic characteristics of the Internet industry and combining Internet enterprise competitiveness, and it is directly related to human resource of a number of factors and applies the AHP method to establish the Internet enterprise human resources management quality and the effect of the evaluation model. Finally, an IoT enterprise is taken as the study object, the human resources management quality assessment model studied in this paper is used to evaluate enterprise's human resources management, and its existing human resources management strategy and its optimization are studied, which provide certain theory support to enhance its market competitiveness.

## 1. Introduction

The Internet of things [1–5] (IoT) is a new thing, which combines computer, Internet, and mobile communication network after the third wave of information industry. The development of huge application prospect has been listed as one of the five emerging strategic industries. Forrester predicted that, according to the research institutions, the IoT industry brought value 30 times larger than the Internet and will form the next $one trillion level communication service.

The IoT that originated in the field of media is the third revolution of information science and technology industry. “If the IoTs get on the bridge of Internet, which through the Internet domain name, we can have instant access to everything anywhere in the world the information. That is, the IoTs with the Internet is equal to earth's wisdom.” IoT is used widely, which can be applied to urban public safety, industrial safety production, environmental monitoring, intelligent transportation, smart home, public health, health monitoring, etc. which help people enjoy a safer and easier life.

IoT can be simply interpreted as follows: by installing information sensing devices, all of them can connect to network, for easy identification and management.

**Figure 1:**
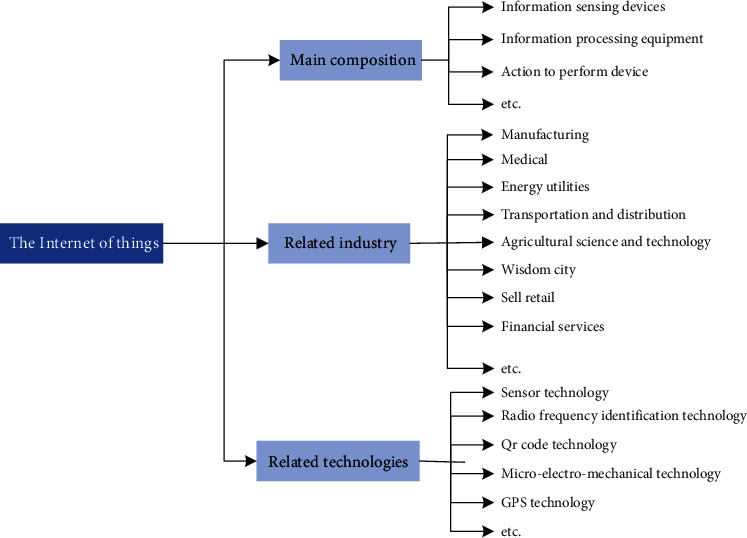
Basic information diagram of IoT.

Figure 1 shows basic information diagram of IoT.

Figure 1 shows that the IoT industry is as follows:

1. The basic situation of the main composition of information sensing and networking devices is used to sense the change of external environment, the information processing equipment, and action execution equipment.
2. The IoT industry mainly [6–10] includes (1) manufacturing: the next generation of the industrial revolution has begun, and this rise is driven by the IoT. Products and equipment have feedback data in the process of using; manufacturing enterprise can make use of the IoT technology; data for risk assessment is used to measure enterprise management risk, protect the company's assets, and improve employees' safety; better access to the customer preferences and use information, such as the behavior, can provide support for subsequent product development; it can improve the reliability of the product and the product performance and service. (2) The medical industry: through the IoT, the patient can choose home for treatment. Medical personnel and equipment can carry on the track of the patient through the IoT, not only for the improvement of the existing conditions, but also for timely response. The recognition of health monitoring and condition will be more quickly than before. IoT technology application in the medical industry can realize the automation of logistics part and shorten the process of logistics. It can also be Unicom Internet information, which will enrich the quality of medical services and diversification. For example, the baby monitor markets collect information and even mobile monitoring mainly through continuous video and audio equipment, but it is very easy to cause false alarms. While, application of the IoTs will pulse the feedback of baby blood oxygen quantitative measurement saturation, which can timely remind parents to mater baby stopped breathing emergency suddenly on mobile phones. (3) Energy and utilities: IoT can supply more energy source and demand; it also can realize the clean integration of renewable energy to generate electricity. From the Internet, people can acquire almost real-time understanding of the equipment information, thereby reducing the effects of temporary power outage. (4) Transport and distribution: IoT trajectory tracking increased to a whole new level. It enables all the parameters to be recorded in the process of delivery, which are not only the location, but also the temperature, humidity, vibration, tilt, and so on. So, the driver and equipment comprehensive monitoring greatly improve the safety. In particular, driverless car is closer to the reality. So IoT technology used in transportation and distribution industry can result in an improvement of operating performance and risk of foresight and more scientific decision-making for operating personnel providing guidance, bringing new business model for transportation industry, and real-time assessment of risk in the process of transportation and distribution, to better protect the security of the enterprise property and employees to provide reliable information support. (5) The agricultural science and technology: the IoT helps to achieve precision agriculture. It helps to make sowing, irrigation, and fertilizer used more accurately. IoT can monitor soil quality, wind speed, and sunshine. This makes the farmers know what level their crops have grown to. Application of IoT in agriculture can also save resources, reduce cost, and reduce the impact on the environment. Through the sensor data, for example, we could suggest a need to how much irrigation water is more suitable. These predictions suggest saving irrigation water and electricity resources, which can also prevent crop disease, reduce cost, and improve the quality of crops. For example, the IoT can help farmers to manage their farms. Through the use of the application and sensors, the farmers can collect, store, and track data of the farm, including temperature, air quality, energy supply, and feed use, and make all kinds of business be remote observation and management of farm. (6) Wisdom city: All forms of smart cities that can not be finished temporarily. But wisdom is in the construction of city. The IoT can be used to reduce energy use, manage traffic, and increase citizens' security. IoT can help account for half of the total number of global populations and urban residents to make their life easier, cleaner, safer, and more pleasant. (7) Landowners retail: IoT has been changing retail. It can make the store shopping experience more personalized. Application of IoT technology in the retail industry can help sellers' insight into customer preferences and behavior better. (8) Financial services in data driven global financial environment: the IoT is to help improve intelligence, reduce risk, and provide better digital experience. It can be used to calculate the insurance fees, credit analysis for accurate, personalized retail banking experience, and provide customized new products. Pet-name ruby intelligent household: household products are an essential part of every family; they are related to people's life and sort is various, and thus, furniture product market activity is high and the market demand is huge.
3. According to statistics in [11–16], in recent years, 80% of household market are working in the field of intelligent household appliance manufacturings. For example, we are familiar with the sweeping machine, TV, washing machine, air conditioner, and even bicycles, locks, and blood pressure monitors have access to the IoT. Some experts predict that, in the future, in 10 years, the IoT can be widespread and will be widely used in intelligent transportation, environmental protection, government, public security and peace, household, intelligent fire control, industrial monitoring, the old man care, personal health, and other fields, giving birth to a scale of trillions of dollars of high-tech market.
4. The IoT technology mainly includes (1) sensor technology, which is sensing technology with computer technology and communication technology and is known as the three main technologies of information technology. From the point of view of bionics, if the computer is regarded as a “brain” of processing and identification information, the communication system is regarded as a “nervous system” of passing information, abd then the sensor is “sense organs.” The microwireless sensor technology and sensor network are the IoT; this component is perception layer of important technical means. (2) Radio frequency identification (RFID) is through the radio signals to identify specific targets and to read and write data wireless communication technology. At home, RFID has been in the ID card, electronic toll collection system, and logistics management, and other fields have a wide range of applications. RFID technology application market is mature, label cost is low, but RFID generally has no data acquisition function, e.g., how to identify items and properties of storage, and limited application in metal and liquid environment; RFID technology belongs to IoT information acquisition technology. (3) QR Code (quick response technology, QR technology for short) is popular in recent years; it is a mobile intelligent device of a kind of coding way; then the traditional Bar Code Bar Code can store more information and more complex data form, and its application scope is more extensive. (4) MEMS: microelectromechanical system refers to the use of large-scale integrated circuit manufacturing technology. MEMS technology belongs to Internet layer information acquisition technology. (5) GPS technology: GPS technology is also called the global positioning system; it is a land, sea, and air omnidirectional real-time three-dimensional navigation and positioning capability of a new generation of satellite navigation and positioning system. As mobile sensing technology, GPS is an IoT extending to the moving object collection and important technology of moving object information, but it is also the important technology of intelligent logistics and intelligent transportation. It is the core technology of information convergence layer: sensor networks, ad hoc network, LAN, and WAN technology.

From the above introduction, the IoT technology is something about application of Internet technology in daily life, which connects to the Internet and helps people use the article intelligent products, enabling them to better meet people's needs. IoT industry is one of the important fields in the future development of science and technology and the bank industry in the human resources management strategy and its optimization is to enhance the bank industry in the enterprise market competitiveness and improve the development potential of one of the important means. This article plans to present the human resources management strategies of IoT industry as the research object; through analyzing the characteristics of the IoT industry human resources, the research uses the AHP method to evaluate Internet enterprise human resources management strategy and combines the multiobjective genetic algorithm for IoT enterprise human resources management strategy optimization. In the end, an IoT enterprise is set as research object, and we present the application of the theory research results of this paper to guide the optimization of the enterprise human resources management strategy.

## 2. Related Works

Fusion rapidly in the world economy and science and technology continue to face fierce competition under the background of market economy; the firms desire the talent more than ever at any time in history; as an important strategic factor, human resource has become the key of enterprise development [17–21] which decide the fate of the enterprise, and other strategic factors, such as resources, knowledge, technology, and main channel need through the participation of human resources to achieve its value. For scientific and effective human resources management, talent guarantee for enterprise development to provide basics, crucially for enterprises in the incentive market competition to win the development of kinetic energy and intellectual support continuing without end, is the most important and fundamental guarantee of sustainable development.

### 2.1. Related Theory about Human Resources Management

Human resource management is used to help a company to achieve certain goals, by adopting a series of planned and systematic human resources development and management, to subordinate staff to select and use, training, evaluation and rewards and punishments, etc. They are a series of management activities. Human resource concept was first presented in 1919 by John R Kang Mons, in his book *Industry Credibility* for the first time.

**Figure 2:**
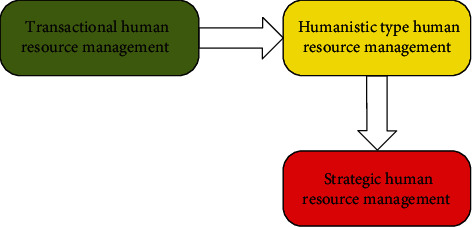
Evolution of the human resources management theory.

Now, however, what we call human resources has been proposed by John R Kang Mons, which had a bigger difference. We now call the human resources concept as the famous American scholar Peter Drucker, who thinks that the human resource is the most precious resources, is unique in all, can promote the development of the enterprise resources, is one of the highest statuses, and has tremendous development potential. The evolution of human resource management mainly experienced three stages [21, 22]: transactional, personnel management, to the humanistic model of human resource management, and strategic human resource management (as shown in Figure 2).

Figure 2 shows that human resources management of the above three stages is progressive transformation; it also exists in the enterprise in the human resources management at the same time. Transactional human resource management is mainly focused on the handling of the “content,” mainly handling of employee files, such as lack of incentive to people. Then retain transactional work of personnel management at the same time; increase the job analysis, salary incentive, and the employees' costs (it is a type of enterprise resources), actively mobilize enthusiasm and creativity of staff, care for staff development, and actively help employees and meet their needs—this is transactional human resource management to people-oriented human resources management transformation process. With the advent of the era of knowledge economy, the popularization of Internet and rapid information transfer, the competition of the market economy has increased, and the humanistic model of human resource management cannot meet the needs of the new situation and started to participate in the enterprise human resources management strategy, so the strategic oriented human resource management arises at the historic moment.

**Figure 3:**
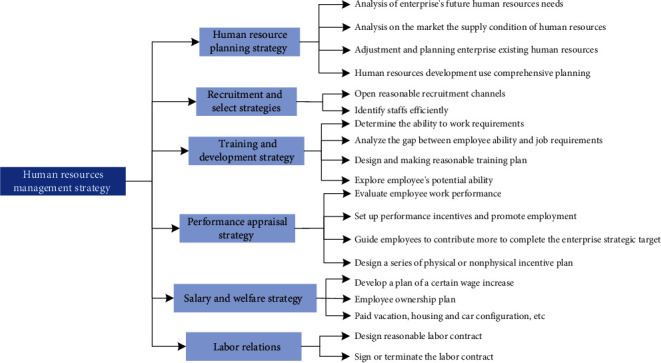
Major means of human resource management.

Human resources management strategy is the operation basis and guiding theory for human resources management of an enterprise. The major means of human resource management strategy is shown in Figure 3.

Figure 3 shows that human resource management mainly includes six types: human resource planning strategy, recruitment and selection strategy, training and development strategy, performance appraisal strategy, salary, benefits strategy, and labor relations strategy.

1. Human resource planning strategy is the enterprise strategic planning as the guide, through the analysis of the human resources of enterprise future human resources demand and market supply situation, carries on the reasonable forecast, adjustment, and planning for the enterprise existing human resources to ensure the comprehensive plan of corporate goals. Human resource planning is the basis for other human resource management practices and is the foundation of the whole human resource management.
2. Recruitment and selection strategy is according to the requirement of the enterprise human resource planning and job analysis, through a reasonable recruitment channels and efficient personnel selection process or a series of process of finding the right people for the enterprise. By methods of vacancy announcement and rehiring priority, selection of internal candidates can also be done through the Internet, advertising, and the university campus to attract a large number of talents for the enterprise. According to the cognitive ability tests, personality and interest test, situational judgment test, no leadership group discussion, management game, background investigation, and a series of method, the right person is selected for the enterprise.
3. Training and development strategy is the ability to dig and cultivate the ability of employees according to the job requirements and staff ability gap analysis, through the design and making of reasonable training plan, in order to improve employees' skills. Before training, enterprise must investigate employee training needs, targeted design training program, and relate to the organization the actual business requirements and enterprise development strategy needs, combined with a variety of training methods to increase staff skills, in order to realize the improvement of organizational performance.
4. Performance appraisal strategy refers to the enterprise in order to effectively motivate employees, according to the requirements of the business goals, and achieve the performance of the standard and adopt the scientific method, the employee's work behavior, and work performance of the process of evaluation and feedback. No matter what kind of enterprises to adopt performance evaluation tools, ultimately they should be able to pay for the staff and provide the reference for the decision-making of post promotion, etc., to staff, illustrate some of the important performance indicators related to business goals, and timely correct the defects of performance plan.
5. The salary and welfare strategy for the enterprise is to keep good employees and adopt a series of material or material incentive plans, like a higher competitive base salary than those in the same industry, employee ownership plan, paid vacation, housing and car configuration, etc. The salary and welfare strategy is most closely associated with personal interests of staff not only to meet basic needs, but also to have certain incentive and maintain staff efficiency, retaining key talent for the enterprise.
6. The labor relations strategy is a method to achieve stable enterprise staff. A way to sign labor contract is to determine the employment relationship between employees and enterprises. Once a contract is signed by the enterprise, employees have certain constraints. Human resource management mode changes over time, and the different enterprise human resources mode in different periods is not fixed. There is no best human resource management strategy that is suitable for all enterprises. The most suitable strategy is to consider the specific combination of enterprise strategic needs and enterprise's actual situation; the human resource management considers multiple factors, to determine the most suitable human resources management strategy.

### 2.2. Introduction about the AHP Theory

In the 1980s, Analytic Hierarchy Process (AHP) was put forward by Professor Say; it is a multilevel, multiobjective scheme of comprehensive comparison method. Its basic principle is as follows: first of all, find out the various factors influencing the decision making, and according to the subordinate relations between them, these factors are set in several levels from high to low; this process is called class hierarchy structure. Then, several leading experts are invited to compare the weight of each factor, and mathematical algorithm is used to calculate the weight of each factor. AHP method is a method of quantitative analysis of qualitative problem; it has the advantages of being simple, flexible, and practical. AHP method is characterized by the complex problem of various factors and is divided into interconnected orderly level and streamline and can better deal with the human resources management strategy optimization problem in the Internet of things industry.

It can be expressed as a mathematical formula and determine the weights of factors with level of judgment matrix *A*.

(1) $$\begin{array}{c} A_{\mathrm{n\times n}}={\left(a_{ij}\right)}_{n\times n}=\left[\begin{array}{cccc} a_{11} & a_{12} & \cdots & a_{1n}\\ a_{21} & a_{22} & \cdots & a_{n2}\\ \vdots & \vdots & \vdots & \vdots \\ a_{n1} & a_{n2} & \cdots & a_{nn} \end{array}\right], \end{array}$$

wherein *a*_*ij*_ represents the importance of the index *i* compared to index *j*. Its specific value is shown in Table 1. The relationship between *a*_*ij*_ and *a*_*ji*_ is as shown in the following formula:

(2) $$\begin{array}{c} a_{ji}=\frac{1}{a_{ij}}. \end{array}$$

Index of each layer is importance weights of experts confirmed, and single sorting and hierarchical total sorts are filled in. Among them, the single sort refers to every judgment matrix in the relative weight of each index for its standards. The steps are as follows: calculating weight to calculate the judgment matrix of the maximum characteristic root and corresponding eigenvectors, and the largest eigenvalue of the matrix is by solving consistency corresponding eigenvectors as weight vector.

(1) Calculating the elements of judgment matrix every line of products *M*_*i*_:
  (3) $$\begin{array}{c} M_{i}=\prod_{j=1}^{n}a_{ij},\;\left(i=1,2,\ldots ,n\right). \end{array}$$
(2) Calculate the *n*th root of each *M*_*i*_:
  (4) $$\begin{array}{c} {\bar{W}}_{i}=\sqrt[{n}]{M_{i}}. \end{array}$$
(3) The characteristic vector normalization processing obtains the corresponding weight coefficient:
  (5) $$\begin{array}{c} W_{i}=\frac{{\bar{W}}_{i}}{\sum_{i=1}^{n}{\bar{W}}_{i}}. \end{array}$$
(4) Consistency check; the purpose of the judgment matrix constructed is reasonable; calculate the maximum characteristic root judgment matrix:
  (6) $$\begin{array}{c} \mathrm{AX}=\lambda_{\max}X, \end{array}$$
wherein *X* is the judgment matrix characteristic root *λ*_max_ which corresponds to the largest eigenvector.
  (7) $$\begin{array}{c} \lambda_{\max}=\frac{1}{n}\sum_{i=1}^{n}\frac{{\left(BW\right)}_{i}}{W_{i}}. \end{array}$$
(5) Computing consistency evaluation index:
  (8) $$\begin{array}{c} CI=\frac{\lambda_{\max}-n}{n-1}. \end{array}$$
(6) Calculate the consistency ratio:
  (9) $$\begin{array}{c} CR=\frac{CI}{RI}. \end{array}$$
In formula (9), *RI* values are as shown in Table 2.
Combining calculation formula (9) and Table 2, the consistency ratio of *CR*, if *CR* < 0.1, is that judgment matrix satisfies the requirement of consistency or needs to adjust the judgment value and through the consistency check.
(7) Computing hierarchy total sorts: it refers to the layer relative to the total target weight coefficient, and the importance of targets at the same time is needed to check the consistency of the combination. Calculation formula is
  (10) $$\begin{array}{c} \text{CRR}=\frac{a_{1}{CI}_{1}+a_{2}{CI}_{2}+\cdots +a_{m}{CI}_{m}}{a_{1}{RI}_{1}+a_{2}{RI}_{2}+\cdots +a_{m}{RI}_{m}}. \end{array}$$
If the *CRR* <0.1, explain level total sorts through the consistency check.

### 2.3. Analysis on the Current Situation of Human Resource Management in IoT Industry

According to Section 1 of this article, introduction about the IoT, the IoT is to point to all kinds of information sensing devices, such as radio frequency identification device, infrared sensors, global positioning system (GPS), and the laser scanner device combined with the Internet and form a huge network. In this network, considering all items with an Internet connection, the system can automatically and in real time identify, locate, track, monitor, and set out the corresponding event. The Internet is an important direction of future development of science and technology; it can help achieve organic combination of the world, human society, and things which can make human in more detail and dynamic management of production and life reach the state of “wisdom,” improve the level of resource utilization and productivity, improve the relationship between human and nature, and improve the ability of the informatization of society as a whole. The IoT industry has a large network and application wide social benefits and characteristics.

At present, the IoT industry chain has been gradually formed; at the same time, the IoT industry human resources division of labor is relatively clear. All aspects of the development of the IoT industry played a positive role in promoting. Emerging IoT development is widely favored by the society. People are also actively participating in and promoting the development of the IoT industry, but also existing Internet management synergy is poorer, highly complex, and insufficient. These deficiencies are also embodied in the greater demand for talent, the high overall cost, the industrial chain of each link of the enterprise relative dispersion, etc.

## 3. An Optimization Example of an IoT Industry's Human Resource Management Strategy

### 3.1. Introduction for an IoT Industry

The IoT company sets “science and technology to create high quality life” as the vision; it is committed to the city and wisdom in the community of intelligent security systems, intelligent management information platform, smart home, intelligent building systems, and green logistics distribution system, which are integrated into the all-in-one, maximizing resource advantage.

### 3.2. Human Resources Situation of an IoT Industry

At present, the Internet enterprise manpower management focuses on how to allocate staff use; stress that gives priority to the personnel, personnel work flexibility, and initiative is small. The biggest characteristic of a personnel management model is high stability, but with modern human resource management it is different. Modern human resources management adheres to the principle of paying equal attention to the use and development, emphasizes the people-centered, thinks HR has the reproducibility and the motility, emphasizes the implementation of organizational goals, and realizes the all-round development of individuals. The key is to inspire people's energy, develop a potential, and make the person work positively, actively, and creatively.

On the value of enterprise, human resources management strategy can be combined with enterprise characteristics affecting its score several key indicators of human resources management. *F*_*hr*_ represents the human resources management strategy score of the IoT enterprise which can be represented as

(11) $$\begin{array}{c} F_{hr}=\sum_{i=1}^{k}K_{i}\times f_{i}, \end{array}$$

wherein *k* is the key to the evaluation of enterprise human resources management strategies score index number; *K*_*i*_, *f*_*i*_ are the key indicators of weight and I score function, respectively.

According to Section 3.1, introduce something connected to the enterprise and the characteristics of the human resources management strategy, determine the impact of its human resources management strategies score, and the key indicators are performance indicators completion, innovation, research and development ability, stability, and business expansion ability. Through market research and consulting, experts concluded that slightly important performance indicators to complete the degree in innovation research and development ability are very important for enterprise stability and are extremely important to expand capacity. Innovative research and development ability in enterprise stability is obviously important to expand capacity. Stability is important in business expansion ability. This results in the Internet of enterprise human resources management strategy evaluation judgment matrix **A**_**hr**_ of key objective weights, which is as follows:

(12) $$\begin{array}{c} A_{\mathrm{hr}}=\left[\begin{array}{cccc} 1 & 3 & 7 & 9\\ \frac{1}{3} & 1 & 5 & 7\\ \frac{1}{7} & \frac{1}{5} & 1 & 5\\ \frac{1}{9} & \frac{1}{7} & \frac{1}{5} & 1 \end{array}\right]. \end{array}$$

Section 2.2 introduces the AHP method which is applied to these key indicators of important degree of sorting; get the weight of each key indicator for **K**:

(13) $$\begin{array}{c} K=\left(\begin{array}{c} 0.5747\\ 0.2882\\ 0.0994\\ 0.0378 \end{array}\right). \end{array}$$

### 3.3. Optimization for the Human Resources Management Strategy of an IoT Enterprise

**Figure 4:**
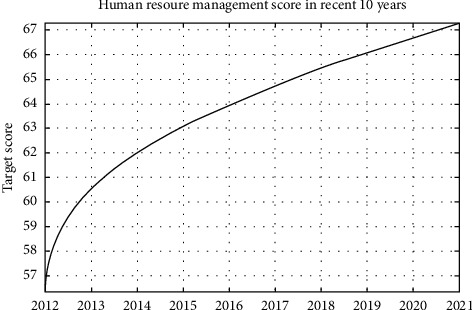
Target score of the enterprise human resources management strategy in nearly 10 years.

We previously introduced something connected to the enterprise and its characteristics of human resources management strategy and determined the key to the enterprise human resources management strategy evaluation index and its weights. the scores of IoTs enterprise human resources management strategy can be calculated; (11)–(13) the scores of IoTs enterprise human resources management strategy can be calculated by analyzing the enterprise, the key indicators of nearly 10 years of human resources management strategy are concluded. Target score of the enterprise human resources management strategy in nearly 10 years is shown in Figure 4.

Figure 4 shows that the IoT in recent 10 years enterprise human resources management strategy improved year by year, but the overall level has been at the lower end and needs to be optimized to improve.

In formula (11), *f*_*i*_ is as follows:

(14) $$\begin{array}{c} f_{i}=f\left(\begin{array}{cccc} x_{1} & x_{2} & \cdots & \begin{array}{cc} x_{j} & \cdots \end{array} \end{array}\right), \end{array}$$

wherein *Xj* is the *j*th factor affecting the *i*th human resources evaluation indicators.

By the analysis of the enterprise in nearly 10 years of human resource management and enterprise operation, the influence on the score of human resource management is more conspicuous for several factors such as material reward, performance bonus plan, employee capacity development training, and employee promotion system.

**Figure 5:**
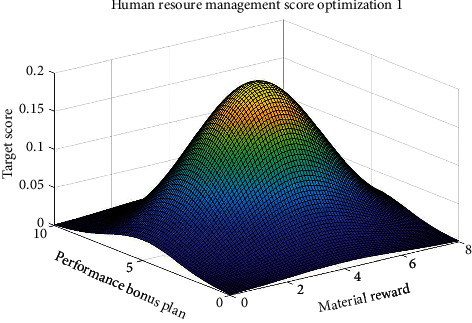
Considering material rewards and performance bonus plan of human resources management strategy optimization.

**Figure 6:**
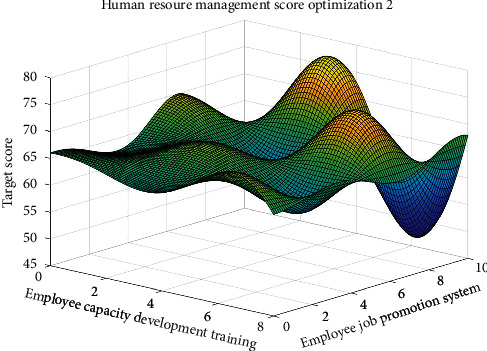
Considering ability to expand training and employee promotion system of human resources management strategy optimization.

Figures 5 and 6 show the four key factors of enterprise human resources management strategy optimization process.

Figures 5 and 6, respectively, show the considering material rewards and performance bonus plan of human resources management strategy optimization process and considering employees' ability to expand training and staff promotion system of human resources management strategy optimization process. Figures 5 and 6 show that the four key factors influencing the enterprise human resources management strategy evaluation are nonlinear, but there are some rules to determine enterprise human resources management strategy. Therefore, when making human resource management strategy in the enterprise, some key factors should be considered, optimizing human resources management strategy as far as possible, to improve the competitiveness of the enterprises.

**Figure 7:**
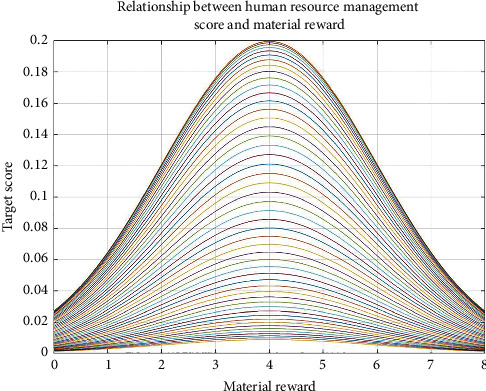
Relationship between human resource management score and material reward.

**Figure 8:**
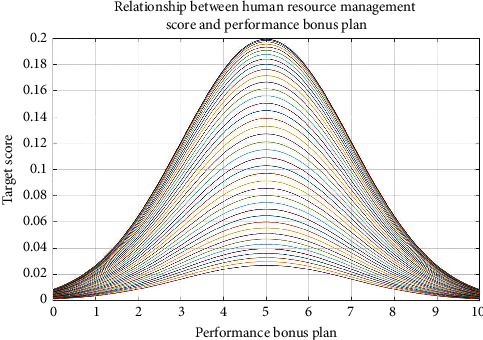
Relationship between human resource management score and performance bonus plan.

**Figure 9:**
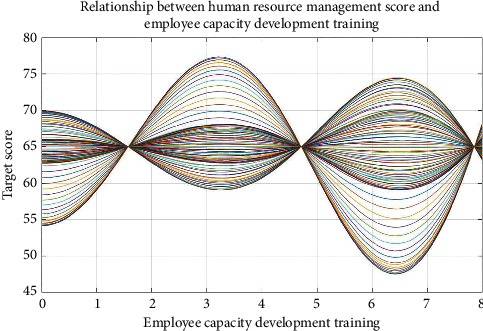
Relationship between human resource management score and employee capacity development training.

**Figure 10:**
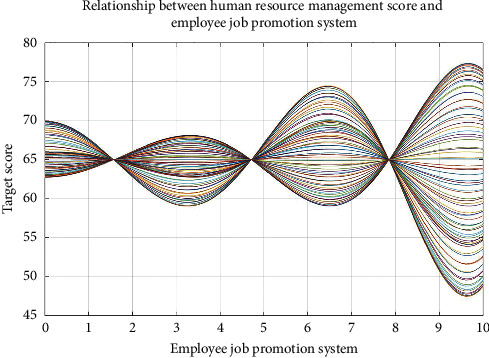
Relationship between human resource management score and employee job promotion system.

Figures 7–10, respectively, show the material reward, the performance bonus plan, the employee capacity development training, and employee job promotion system of the four key factors and the relationship between enterprise human resources management strategies scores.

Figures 7–10 show that (1) the material reward, the performance bonus plan, the employee capacity development training, and employee job promotion system of the four key factors influence on the enterprise human resources management strategy evaluation effect is different; (2) improving performance is not necessarily a key factor to the enterprise human resources management strategy; and (3) the factors that affect the enterprise human resources management strategy evaluation include not only the above four key factors; if you want to more accurately improve enterprise human resources management level, increase the multiple factors analysis.

## 4. Conclusions

This article expounds the IoT, human resources strategy, and AHP and other related concepts, characteristics, and principles and analyses the current situation of the development of the IoT industry and its present situation of human resource management. Studies found that the IoT industry focus of human resource management is how to allocate employees use, with excessive emphasis on work as the center, staff the flexibility and initiative are small, the characteristics of human resources management are not able to satisfy the Internet industry, and to a certain extent, they restrict the development of the IoT industry.

The article presents, through analyzing the characteristics of the IoT industry development and its internal, something connected to the enterprise as the research object, combins the enterprise management situation in nearly 10 years, determines the decision of the enterprise human resources management strategy evaluation of four key indicators, and applies the AHP method to calculate the weights of these four key indicators. On the basis of the above, we study the effect of the above four key indicators of the four main factors (material reward, the performance bonus plan, the employee capacity development training, and employee job promotion system), to build the Internet of enterprise human resources management evaluation model and study and optimize the four main factors on the enterprise human resources management strategy; the research conclusion can provide Internet enterprise optimizing human resources management strategy to provide theoretical guidance.

## Figures and Tables

**Table 1:** The judgment matrix scale method.

Scale	Definition
1	Two factors are equally important
3	The *i*th index is slightly more important than the *j*th index
5	The *i*th index is more important than the *j*th index
7	The *i*th index is obviously more important than the *j*th index
9	The *i*th index is extremely more important than the *j*th index
2, 4, 6, 8	Between the two adjacent judgment values

**Table 2:** The value of *RI*.

*n*	1	2	3	4	5	6	7	8	9	10	11	12
*RI*	0	0	0.58	0.9	1.12	1.24	1.36	1.41	1.46	1.49	1.52	1.64

## Data Availability

The data used to support the findings of this study are available from the corresponding author upon request.
